# Surface Modification Techniques to Produce Micro/Nano-scale Topographies on Ti-Based Implant Surfaces for Improved Osseointegration

**DOI:** 10.3389/fbioe.2022.835008

**Published:** 2022-03-25

**Authors:** Chuang Hou, Jing An, Duoyi Zhao, Xiao Ma, Weilin Zhang, Wei Zhao, Meng Wu, Zhiyu Zhang, Fusheng Yuan

**Affiliations:** ^1^ Department of Orthopedics, The Fourth Affiliated Hospital of China Medical University, Shenyang, China; ^2^ Nursing Teaching and Research Department, The Fourth Affiliated Hospital of China Medical University, Shenyang, China

**Keywords:** surface modification, titanium-based, nano-scale, micro-scale, implant

## Abstract

Titanium and titanium alloys are used as artificial bone substitutes due to the good mechanical properties and biocompatibility, and are widely applied in the treatment of bone defects in clinic. However, Pure titanium has stress shielding effect on bone, and the effect of titanium-based materials on promoting bone healing is not significant. To solve this problem, several studies have proposed that the surface of titanium-based implants can be modified to generate micro or nano structures and improve mechanical properties, which will have positive effects on bone healing. This article reviews the application and characteristics of several titanium processing methods, and explores the effects of different technologies on the surface characteristics, mechanical properties, cell behavior and osseointegration. The future research prospects in this field and the characteristics of ideal titanium-based implants are proposed.

## Introduction

Bone is a mineralized connecting tissue composed of nano/micro-textured extracellular matrix, bone cells, and many biological functional elements. It is mainly composed of collagen in the bone matrix to provide tensile strength, and Calcium salts between bone cells are used as its main mineral components to provide compressive strength (Wei et al., 2020), that is why the skeletal system can provide support and protection for the tissues and organs of the body. Bone defect is a disease in which the integrity of the bone structure is destroyed due to congenital or acquired reasons (Bray et al., 2018), and it tends to occur near the joints, especially the tibia, proximal humerus, femur, and distal radius, and it is most common in patients with comminuted fractures, patients with infections in fractures, and patients with bone tumors in the metaphysis of long bones (de Melo Pereira and Habibovic, 2018). Approximately 20 million patients worldwide lose bone tissue due to various diseases every year, however there are many reasons for that, such as comminuted fractures, large bone tissue defects caused by open fractures, osteonecrosis caused by inflammation, bone loss caused by bone tumor resection, bone infarction or large bone defect caused by bone avascular necrosis, etc.(Li et al., 2015). Symptoms such as local pain caused by bone defects, abnormal movement of limbs, and functional limitation will cause severe economic burden and reduced quality of life for patients. More importantly, serious complications include purulent osteomyelitis, osteonecrosis and bone marrow cavitation, and cause pathological fractures, amputations and even death to the patient (Agarwal and Garcia, 2015). Therefore, the treatment of bone defects is extremely important.

The bone repair process involves a variety of cells in the bone microenvironment, such as dendritic cells, mesenchymal stem cells, osteoblasts, and endothelial cells. These cells influence and regulate each other to influence bone repair and reconstruction.

Bone defects smaller than the critical size (Mauffrey et al., 2015) (longer than 1–3 cm, bone circumference loss more than 50%), the body can generally heal itself, while bone defects exceeding the critical size, However, for bone defects that exceed the critical size, the anatomical location of the defect, the damage of the surrounding tissues and the state of the body must be considered, and because the gap between the broken ends of the bone is too large, it is difficult for the body to heal itself, so it must be treated by surgery (Mauffrey et al., 2015). At present, the clinical treatment of bone defects is mostly based on surgery, and there are various surgical treatment methods, including Ilizarov technology, bone filling technology of autologous or allogeneic bone, filling technology of artificial bone replacement material, etc.(Huang et al., 2016; Hasegawa et al., 2020). However, Ilizarov technology usually affects the treatment effect due to the nonunion of fracture ends and soft tissue incarceration caused by infection and the long treatment time can lead to complications such as pressure sores and infections (Hasegawa et al., 2020). Although autologous bone transplantation is the gold standard for clinical surgical treatment of repairing smaller defect areas, it also has many shortcomings and limitations: including limited autologous bone available, pain at the donor site, and deep donor infection (Huang et al., 2016). Although allogeneic bone transplantation can solve some of the above problems, these donor bone tissues carry the risk of recipient infection, disease transmission and immune response (McNamara et al., 2011).

Metal materials are particularly important in the filling technology as artificial bone substitute materials. After surgical implantation of metal materials, according to the specific surface morphology and chemical composition of the metal materials, biological events such as osseointegration will occur at the bone-implant interface. However, implants with appropriate nano/microstructure morphology and chemical composition will enhance bone repair and bone tissue regeneration. It can be seen that metal materials can more comprehensively solve the above-mentioned problems including infection, immune response and avoiding the use of autologous bone. Nowadays, there are a few clinical applications of metal materials in the treatment of bone defects, but the use of suitable metal materials to treat bone defect repair will be an important research and development direction in the future.

**SCHEME 1:**
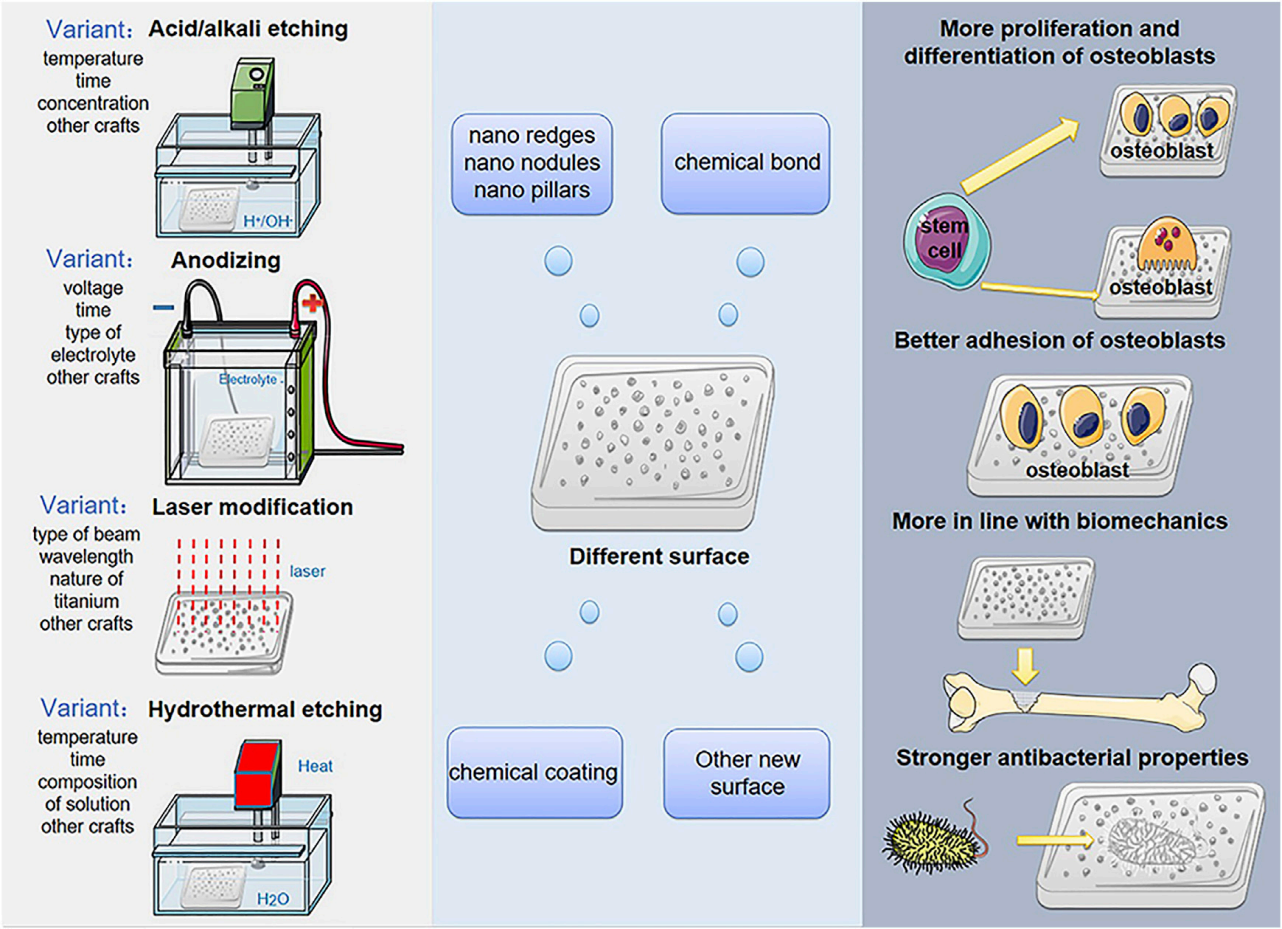
Surface modification treatments produce different surfaces of titanium-based implant in order to promote cell proliferation, differentiation, adhesion, biomechanical and antibacterial properties.

Titanium and its alloys have excellent biocompatibility, low elasticity, and good corrosion resistance. These special characteristics have led to wide spread use as artificial bone substitute materials (Hansson and Norton, 1999; Zhao et al., 2010; Clainche et al., 2020). With the vigorous development of metal material engineering technology and tissue engineering technology, artificial bone implants made of titanium alloys have gradually attracted more and more interest from researchers and clinicians as alternative materials for the treatment of bone defects. Just as technologies such as acid/alkali etching, anodizing, laser modification and hydrothermal etching will give titanium and its alloys different surface microstructures (Sharma et al., 2015; Kaur and Singh, 2019; Vishnu et al., 2019; Wozniak et al., 2020) which will influence the differentiation level of bone osteoblasts, the level of adhesion of bone cells, the strength of antibacterial properties and the level of mineralization, while these cellular-level influences determine the rate of osseointegration and the strength of bone defect repair, and better osseointegration and the strength of the bone defect after repair is critical to the patient’s prognosis (Jing et al., 2016) (Scheme 1).

This article will focus on the impact of different preparation processes on the surface microenvironment of titanium alloys when titanium-based implants are used as artificial bone substitutes for the treatment of bone defects, and the effects of the titanium-based implants surface microenvironment under this process on the treatment of bone defects. These impact, which in turn represents the therapeutic effect of titanium and its alloys as bone substitute materials for bone grafting to treat bone defects. With the rapid development of modern metal material engineering technology and histological engineering technology at any time, more and more preparation processes for the surface microenvironment of titanium-based implants are emerging in an endless stream, bringing more clinical treatment options and more personalized treatment opportunities for patients. The author made a review for this, and at the same time looked forward to the future research direction, aiming to provide a reference for researchers in the field of bone repair.

## Equal Channel Angular Pressing

Commercial pure titanium (cp-Ti) and Ti6Al4V (Ti64) alloy are the most popular titanium-based materials in orthopedic implants. Ti64 has advantages over cp-Ti because of its higher strength and less prone to fatigue and/or breakage. However, the Al and V contained in the Ti64 alloy are toxic to the human body, and although the content is low, there is a potential biosafety risk (Chappuis et al., 2018). Therefore, several techniques have been developed to improve the mechanical strength of cp-Ti, to make it a valuable substitute for titanium alloys. These include 3D printing porous titanium and severe plastic deformation (SPD). SPD improves the mechanical properties of the material without changing its original shape and size by applying high plastic strain to the material (Attarilar et al., 2019). Equal channel angular pressing (ECAP) is one of the most common processes in SPD. To date, there are few reviews on ECAP improving the performance of orthopedic implants.

According to the Hall-Petch relation, the smaller the grain size value above the critical value, the higher the strength (Machackova, 2020). But plastic deformation is difficult to achieve grain refinement. Therefore, the SPD including ECAP method was developed to achieve this (Estrin et al., 2013). Several studies have shown that the grain size (4.5–110 μm) of untreated cp-Ti is transformed to an average value of 200–500 nm after ECAP, from coarse-grained (CG, micron scale) to ultra-fine grained (UFG, sub-micron scale) class) (Estrin et al., 2009; An et al., 2016; Attarilar et al., 2019; Wu et al., 2019; Asl and Alsaran, 2020). During this process, the fatigue strength of cp-Ti increased by 138.9% (Estrin et al., 2009), the yield strength increased by 141–157.3% (An et al., 2016; Attarilar et al., 2020), the ultimate shear strength increased by 78% (Attarilar et al., 2020), the ultimate shear strength increased by 78% (Attarilar et al., 2020), the ultimate tensile strength increased by 50.9% (Wu et al., 2019) and Vickers hardness increased by 63.4% (An et al., 2016). Meanwhile, UFG surface improves cell adhesion, proliferation and differentiation (Estrin et al., 2009; Park et al., 2009; Estrin et al., 2013; Attarilar et al., 2019). However, the elastic modulus of the material did not change significantly (Wu et al., 2019).

According to the above results, ECAP can significantly increase the strength of cp-Ti and increase the service life of the implant without reducing the biocompatibility. But on the other side, since the elastic modulus of Ti is higher than that of cortical bone, the stress shielding effect of cp-Ti prevents bone healing and leads to implant failure (Yang et al., 2011). However, ECAP doesn’t improve the elastic modulus of cp-Ti. This makes ECAP processed cp-Ti not currently a perfect substitute for TI64 alloy.

## 3D Printing

3D printing (3DP), a branch of additive manufacturing (AM), is a process of producing materials with specific structures from a digital virtual model of a desired object. ASTM committee divides 3DP into 7 types according to different production procedures: vat photo-polymerization (VP); powder-bed fusion (PBF); binder jetting (BJ); material jetting (MJ); material extrusion (MEX); sheet lamination (SL) and directed energy deposition (DED) (Attarilar et al., 2021). Among them, PBF is the most commonly used technique for producing metal implants. During the PBF process, a thin layer of metal powder is selectively melted by a heat source based on CAD slice data in the preferred areas and solidified, then the powder bed is lowered and the procedure is repeated on a new powder layer until complete (Rafi et al., 2013; Attarilar et al., 2021).

Therefore, porous titanium was developed, which can effectively avoid stress shielding with similar bone stiffness, enhance the mechanical properties of scaffolds with suitable porosity, and increase the surface area for loading functional molecules or drugs (Bsat et al., 2015). However, due to the high chemical reactivity of titanium, the control of pore structure by most methods is often limited to the implant surface (Puckett et al., 2010). Therefore, 3DP is used to fabricate porous titanium, as it can precisely obtain porosity with an optimal level (Bsat et al., 2015), and the mechanical properties of the scaffold can be changed by adjusting the pore microstructure inside the scaffold according to different needs (Webster and Ejiofor, 2004).

PBF includes the two most common processing methods, selective laser melting (SLM) and electric beam melting (EBM). SLM uses the laser beam as the heat source, with smaller powder and thinner layer, and is characterized by a high cooling rate; while the EBM uses the electron beam as the heat source, with larger powder and thicker layer, and has faster production process than SLM (Rafi et al., 2013). Furthermore, there are different characteristics on the surfaces of the 3DP titanium samples produced by the two methods. The surface of EBM scaffolds has obvious inhomogeneity and greater roughness (Ginestra et al., 2020). In the study of Ginestra et al. and Ren et al., the roughness Ra of EBM titanium alloy samples is 25.51–51.0 μm, while the Ra fluctuating of SLM titanium alloy samples is 17.4–24.4 μm (Ginestra et al., 2020; Ren et al., 2021). This can be attributed to differences in layer thickness as well as powder particle size (Ginestra et al., 2020). Zhao et al. demonstrated that the EBM scaffold surface is more hydrophilic than the SLM surface, with contact angles of approximately 86° and 101°, respectively (Zhao et al., 2017). Greater roughness and hydrophilicity usually mean better cellular response behavior, but on the other hand, the greater initial surface roughness of 3DP scaffolds often leads to a decrease in mechanical properties. In addition, the two processes produce different microstructures. The SLM surface is usually an α’ martensite microstructure (Rafi et al., 2013), while the EBM surface is dominated by α lamellae (Koike et al., 2011; Rafi et al., 2013).

The corrosion resistance of SLM samples is better than that of SLM samples (Koike et al., 2011; Wang et al., 2016). In two studies, compared with EBM titanium alloy, the yield strength of SLM titanium alloy is increased by about 33 and 11%, and the tensile strength is increased by about 30 and 11%, respectively (Koike et al., 2011; Rafi et al., 2013). In the study by Rafi et al., the fatigue limit of the SLM sample is about 61.8% higher than that of the EBM sample, while the strain at break is 47% lower (Rafi et al., 2013). These results indicate that SLM scaffolds have greater corrosion resistance and strength, while EBM scaffolds have better plasticity. This can be attributed to the α’ martensite microstructure of the SLM samples and the lamellar α phase of the EBM samples. However, in the study by Koike et al., the strain at break shows the opposite trend, namely 6% in SLM and 2% in EBM, which may be due to the higher oxygen content of the Ti64 powder recovered by EBM (Koike et al., 2011).

In the field of orthopaedic implant production, 3DP has some significant advantages compared to other processes: 1. Repeatability and mass production; 2. Low production cost; 3. Unused powder can be recycled for optimal usage rate; 4. Personalized customized materials; 5. Improvement of mechanical properties and corrosion resistance of materials; 6. Easy to sterilize materials during production; 7. Short production cycle. However, there are some problems that limit the use of 3DP. 3DP scaffolds retain unmelted shedding powder during processing (Attarilar et al., 2021), and the surface cannot meet the requirements of promoting osseointegration *in vivo* (Ren et al., 2021), so subsequent surface treatment is usually needed before implantation. Additionally, powders that are recycled multiple times during processing can absorb oxygen due to titanium’s affinity, negatively affecting mechanical properties (Murr et al., 2009).

## Acid/Alkali Treatment and Anodization Treatment

Acid etching is a chemical method of surface modification in which titanium-based implants are immersed in a strong acid solution under certain conditions, including hydrochloric acid (HCl), nitric acid (HNO_3_), sulfuric acid (H_2_SO_4_), hydrofluoric acid (HF), etc. (Zahran et al., 2016; Hung et al., 2017; Lario et al., 2018) Alkali-heat treatment are to first soak the titanium-based material with a strong alkali solution (usually NaOH), and then treat it at high temperature in a heating furnace for a certain period of time (Su et al., 2016; Yang et al., 2018; Tan et al., 2020). Acid etching and alkali-heat treatment are both simple and easy surface modified methods, which can produce micro-scale or nano-scale topography on the titanium-based implant surface, and further affect cell adhesion, migration, proliferation, differentiation and osseointegration (Wang et al., 2020). The combined use of acid etching and sand blasting is Sand-blasted, Large-grit, and Acid-etching (SLA), which is currently the most widely used implant surface treatment method (Li and Wang, 2020; Souza et al., 2020). Alkali-heat treatment can also be combined with micro-scale surface modification such as sandblasting to form a micro-nano hybrid structure (Zhuang et al., 2014; Gao et al., 2021; Hu et al., 2021). Anodization is an electrochemical method of surface modification on metal surfaces. The nano surface structure can be formed by simultaneously performing metal oxide formation and selective dissolution on the surface. The titanium oxide nanostructure formed by anodization has a similar arrangement to that of normal bone tissue collagen fibers (Souza et al., 2019), and the elastic modulus of titanium oxide (about 36–43 GPa) is closer to that of cortical bone (11–30 GPa). For cp-Ti (110–140 GPa), it is more conducive to reducing stress shielding (Souza et al., 2019). In general, anodization is a simple and economical technique, and the formed titanium oxide layer has good corrosion resistance (Yu et al., 2011).

By changing the Acid etching or alkali-heat treatment parameters, such as immersion temperature, duration, acid or alkali concentration, and heating furnace temperature, the surface of different scales or morphologies can be formed (Zhuang et al., 2014; Kato et al., 2015; Fu et al., 2020). A common acid etching treatment is to soak the titanium-based implants in a strong acid solution for 60 min at 60–100°C and ultrasonic vibration (Souza et al., 2019). Common alkali-heat treatment conditions include 5 M alkali solution concentration, 60°C immersion temperature, 5°C/min heating rate in the heating furnace, and 600°C, 1 h heating process (Kato et al., 2015; Kawai et al., 2015; Yang et al., 2018). Acid etching usually forms micro-scale pits and spikes on the surface, while alkali-heat treatment usually forms a nano-scale topography (Zhuang et al., 2014; Yamada et al., 2016; Wang et al., 2020). Nanotubes are a typical topography formed on the surface of titanium-based implant by anodization (Wang et al., 2011; Lv et al., 2015; Yang et al., 2016). The gap between the nanotubes facilitates the transport of signaling molecules (Zhang et al., 2015). By changing the parameters of anodic oxidation, such as voltage, duration, temperature, electrolyte composition and electrolyte fluid dynamics conditions, etc., the size, spacing or degree of order/disorder of the nanotubes can be changed, thereby affecting the cells behavior on the surface of the material (Duvvuru et al., 2019; Gong et al., 2019; Xu Z. et al., 2020; Li et al., 2020).

**FIGURE 1:**
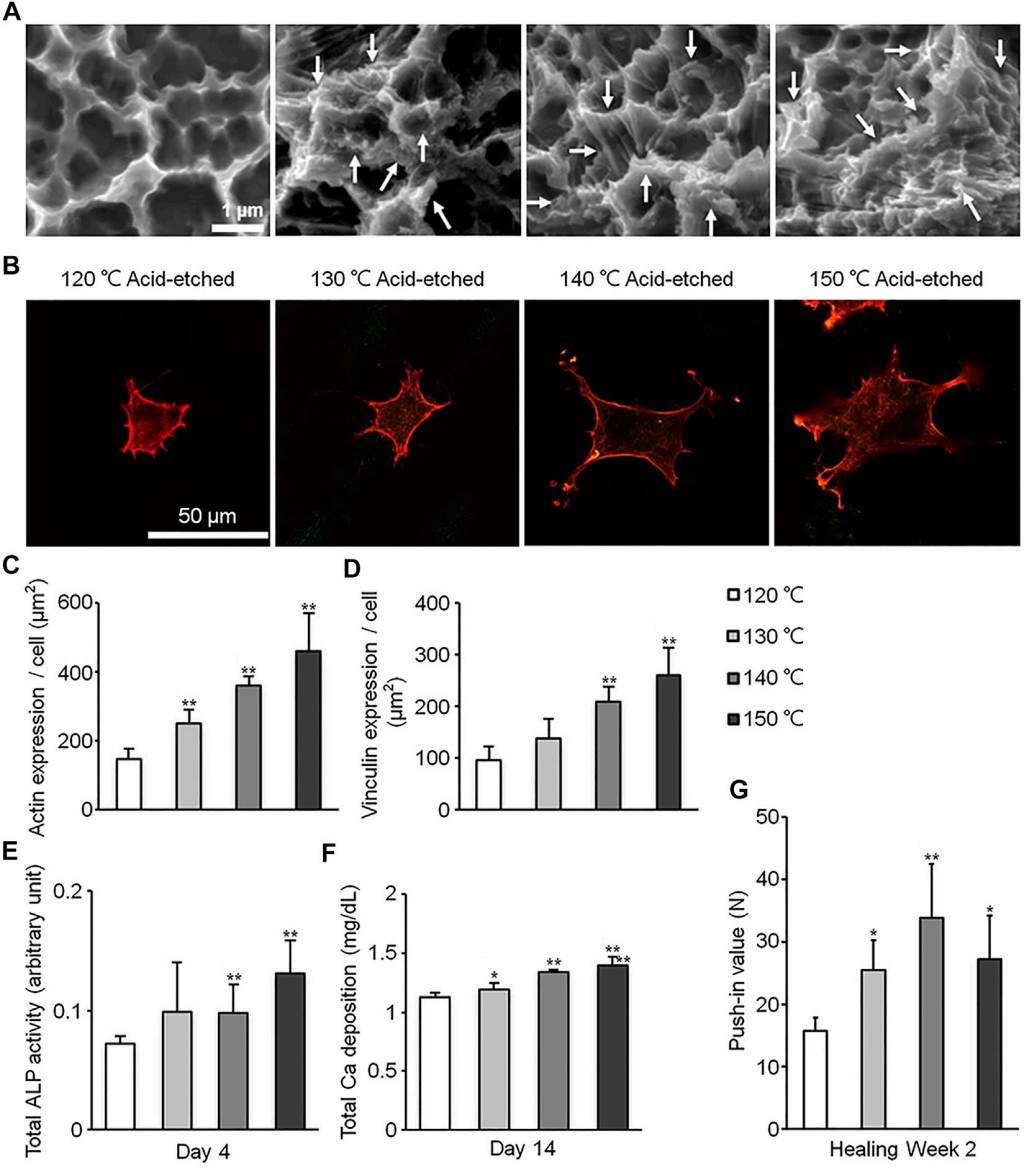
Surfaces of different scales processed through acid etching at different temperature. **(A)** Scanning electron microscopy (SEM) photographs of surfaces in different groups. **(B)** Photographs of cells stained with rhodamine phalloidin. **(C–G)** Quantitative results of actin expression **(C)**, vinculin expression **(D)**, ALP activity **(E)**, Ca deposition **(F)** and push-in value **(G)**. Reprinted with permission from (Hasegawa et al., 2020).

Studies have shown that the microstructure has a size similar to that of cells and bone resorption pits (Huang Y. et al., 2019; Saruta et al., 2019). Therefore, the addition of micron topography on the titanium-based implant surface can promote cell differentiation and mechanical interlocking (Krishna et al., 2007), but may have a negative effect on cell spread and proliferation (Saruta et al., 2019), as cells tend to spread in confined spaces in microgrooves (Harcuba et al., 2012). The nano-scale topography often has obvious promotion effect on cell adhesion, proliferation and differentiation, which may be due to its similar size to the proteins in extracellular matrix and the membrane receptors (Ren et al., 2021). Masakazu Hasegawa et al. produced a new multi-scale hierarchical surface through an acid etching process, which can overcome the negative effect of the micro-scale topography (Figure 1A). Compared with a simple micrometer scale, the area of osteoblasts on the multi-scale surface increased by about 0.5–1 times (Figure 1B), and the expression levels of actin and vinculin increased by about 1–2 times (Figures 1C,D), showing higher spread ability. In addition, alkaline phosphatase (ALP), calcium deposition and osseointegration were also improved (Figures 1E–G). This indicates the nano-scale can reduce the possible negative impact of the micro-scale on cell spreading, and significantly promote the differentiation of osteoblasts and osseointegration (Hasegawa et al., 2020). Zhao et al. and Hao et al. performed anodization on the acid-etched titanium surface, adding nanostructures to the original microstructures. The results showed that the newly added nanostructures increased cell proliferation while retaining the ability of mechanical interlocking and differentiation promoting (Zhao et al., 2010; Hao et al., 2017). Takeshi Ueno et al. produced micro-nano hybrid surface by sand blasting and alkali-heat treatment, which exhibits more pronounced new bone formation and osseointegration than micro-scale surface (Ueno et al., 2011). Naoki Tsukimura et al. found that the microstructure produced by sandblasting treatment increased implant biomechanical strength in the early stage of healing (1–4 weeks), which was manifested as a 70–120% increase in push-in value, compared to the machined surface without microstructure, but did not increase in the late healing period (8 weeks). Further, They added nano-scale features to the sandblasted microstructure surface through alkali-heat treatment. This micro-nano composite surface has been proven to increase the push-in value by 60–100% 1–8 weeks after implantation, compared with the sand blasted microstructure surface, and significant new bone formation on the surface was observed (Tsukimura et al., 2011). These results reflect the synergistic effect of micro- and nano-scale surface topography produced by acid etching, alkali heat and anodization. In recent years, some researchers have found that the Akt signaling pathway is a key factor in the effect of hierarchical micro-nano topography on cellular responses. Studies have shown that cells cultured on hierarchical micro-nano surfaces exhibited better adhesion and osteogenic differentiation capacity as well as activated Akt pathways, compared to cells on smooth, micro- or nano-scale topographical surfaces. This difference was eliminated with the use of Akt-specific inhibitors.

**FIGURE 2:**
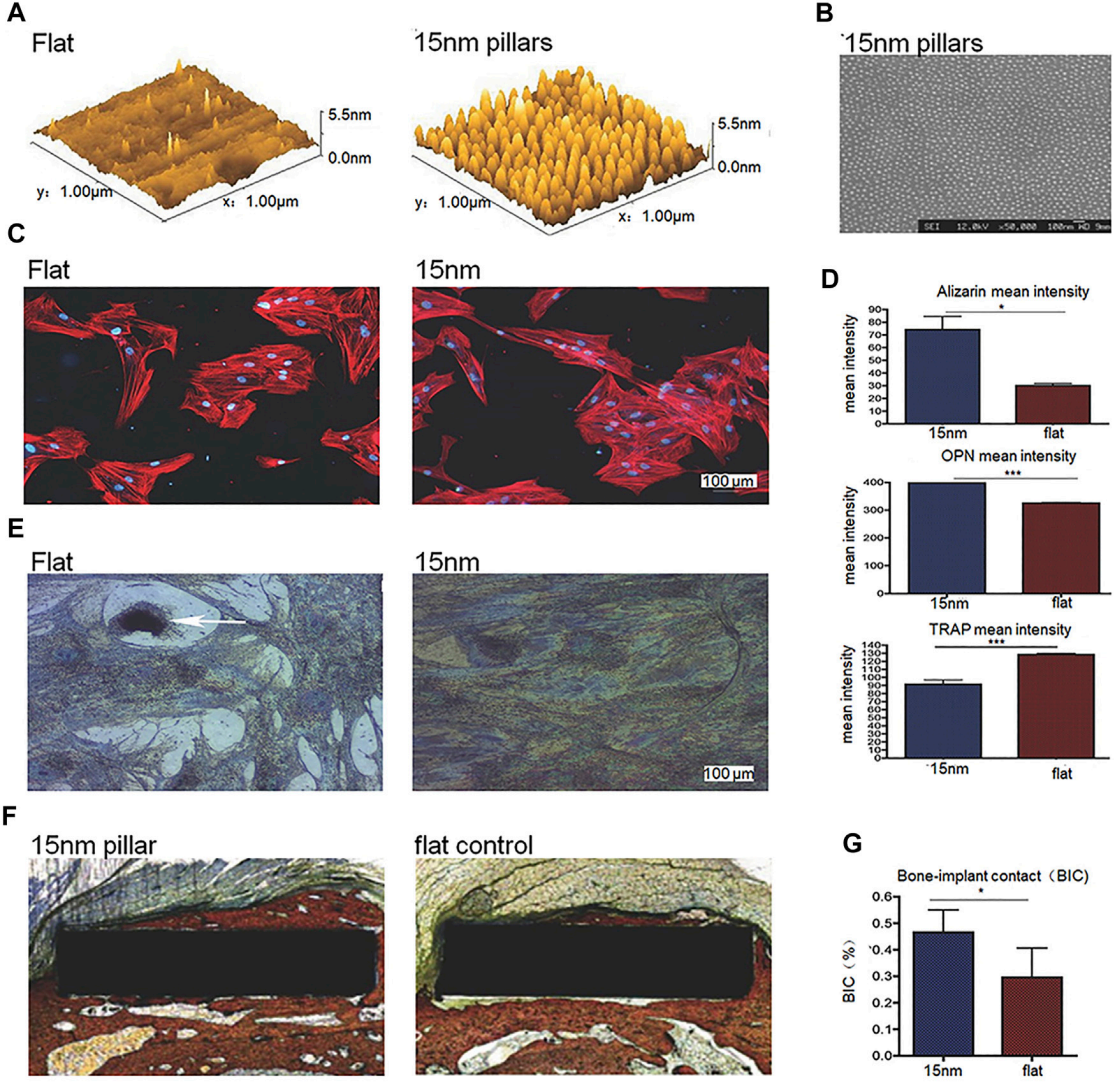
**(A,B)** 3D Atomic Force Microscope (AFM) height images and SEM image of disordered 15 nm nanotube arrays on the titanium surface processed through anodization. **(C)** Immunofluorescent images of cells on surfaces in different groups. **(D)** Mean intensity of alizarin, osteopontin (OPN) and tartrate resistant acid phosphatase (TRAP, indicating osteoclastic activity of the cells) of cells on surfaces in different groups. **(E)** TRAP histochemical images of cells on surfaces in different groups. **(F,G)** Histological staining images and quantitative results of bone-implant contact (BIC). Reprinted with permission from (Silverwood et al., 2016).

Laura E. McNamara et al. used anodizing technology to produce TiO_2_ nanotube sequences with three different heights of 15/55/90 nm on the titanium surface in their research, which have a certain degree of disorder. The results show that, compared to purely polished titanium surface and 55/90 nm high nanotube sequence titanium surface, mesenchymal stem cells on 15 nm nanotube sequence titanium have the highest RUNX2 and osteocalcin expression (McNamara et al., 2011). In addition, Robert K. Silverwood et al. produced disordered 15 nm high nanotube arrays on the titanium surface through anodization (Figures 2A,B), which were proven to increase osteoblasts, reduce osteoclasts and increase osseointegration, compared to polished smooth Ti (Figures 2C–G) (Silverwood et al., 2016). Previous studies have shown that the disordered arrangement (Dalby et al., 2007) or the height below 20 nm of surface nano-scale features is the promoting factor of cell response, compared to ordered arrangement or higher nanostructure height. Therefore, the researches of Laura E. McNamara and Robert K. Silverwood show that it is feasible to use anodizing method to produce nanotubes with a certain disorder and a <20 nm height on the titanium-based implant surface to enhance osteogenic differentiation or osseointegration.

## Laser Modification

Laser modification is a new type of micro-machining surface texture technology that is different from traditional machining technology (Branemark et al., 2011). The thermal effect and photonic effect of the laser are used in laser modification to melt or vaporize the titanium-based implant surface to control the texture and chemical composition of the surface (Biswas et al., 2016; Zhao et al., 2019; Rafiee et al., 2020). Interference is a type of laser processing technology that uses two or more laser beams to form a periodic texture on the surface of the titanium-based implant (Hartjen et al., 2017; Zwahr et al., 2017).

**FIGURE 3:**
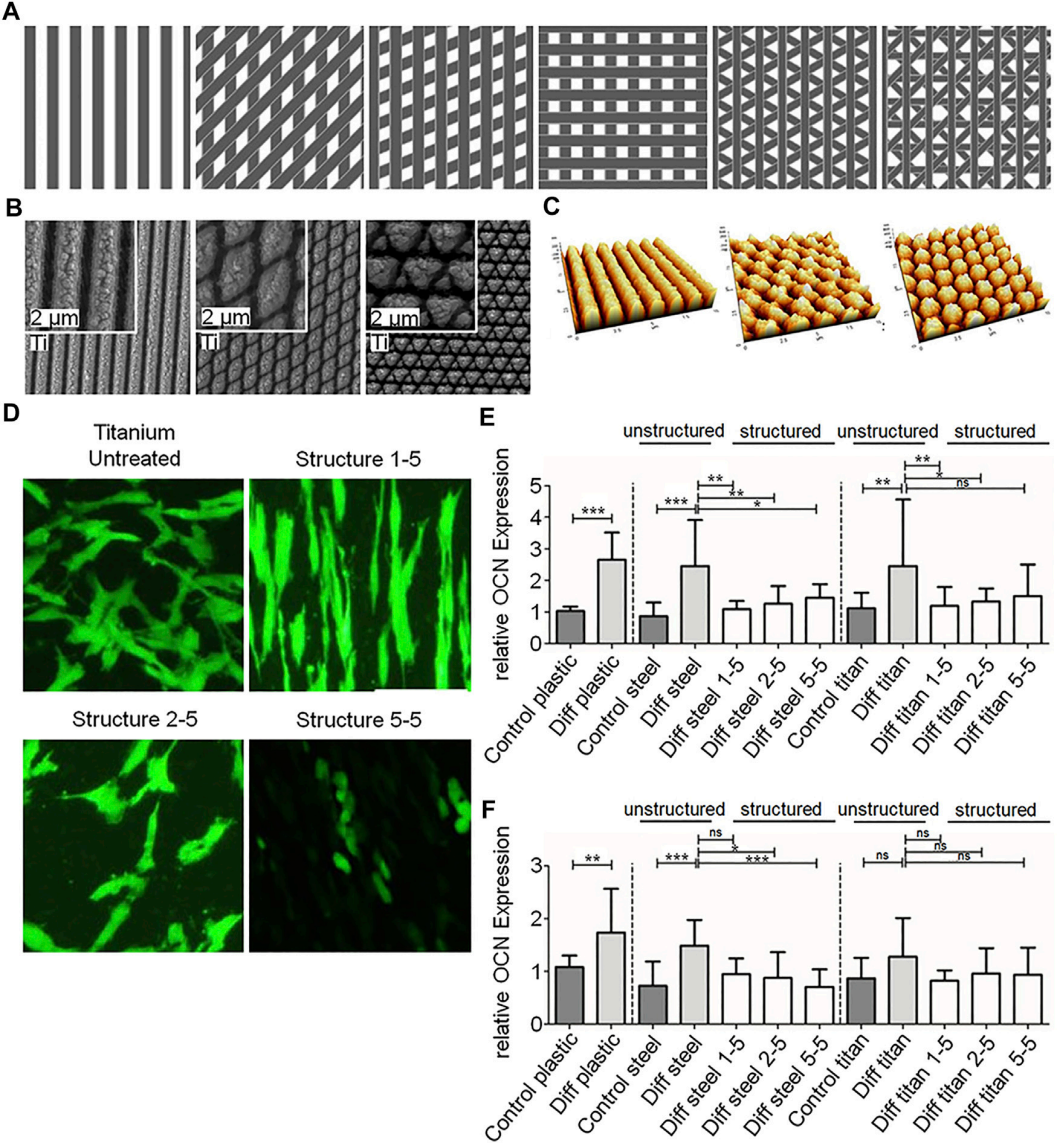
**(A–C)** Schematics, SEM images and AFM images of nano-scale textures with different patterns on laser ablated titanium. **(D)** Morphologies of cells on different nano-scale textures. **(E,F)** Quantitative Results of OCN and RUNX2 expression in different groups. Reprinted with permission from (Boker et al., 2020).

As a kind of micro-mechanical processing, laser modification is a non-contact, fast and highly controllable processing technology compared to chemical modification methods and traditional mechanical processing methods (Matsugaki et al., 2015; Klos et al., 2020; Liu et al., 2020). It is reproducible and environmentally friendly (Ionescu et al., 2018; Wang et al., 2021). More importantly, laser modification can precisely control the location or specific pattern of texture to achieve different purposes. For example, only selectively generating nano-scale textures on the surface of titanium screw thread valleys to increase osseointegration (Shah et al., 2016), or imitating fish scales and shrimp back surfaces to produce specific bionic overlapping textures on the surface of titanium alloys, which can promote the formation of apatite particles, in order to reduce the abrasion of the implant surface and prolong its service life (Xu Y. et al., 2020). Furthermore, Boker et al. fabricated different periodic nanogrid structures on the titanium surface by laser ablation (Figures 3A–C), and found that different patterns had different effects on cell adhesion and differentiation (Figures 3D–F) (Boker et al., 2020). These results show that laser modification is a flexible and accurate surface processing technology.

**FIGURE 4:**
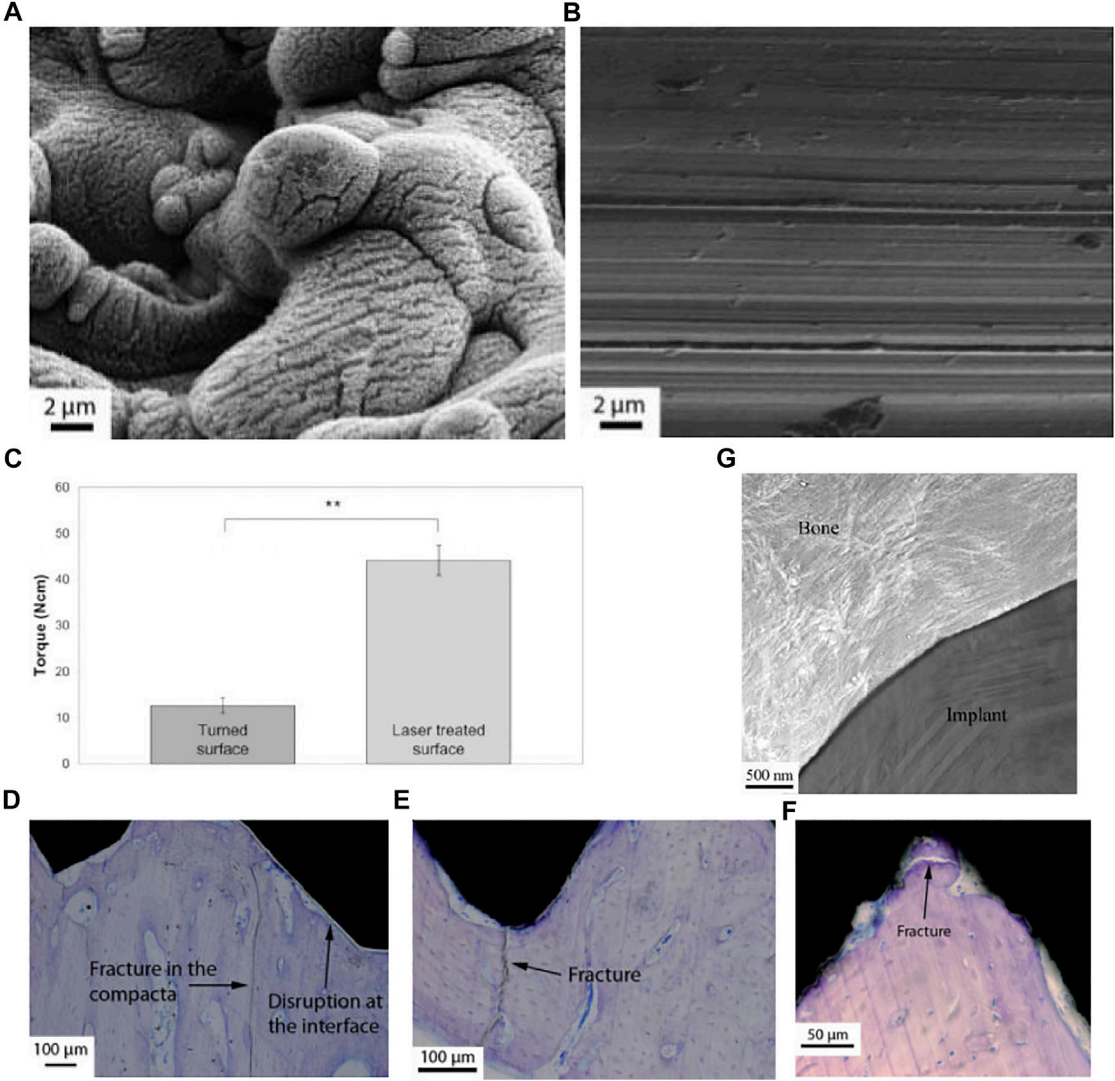
**(A,B)** SEM images of laser-treated surface (left) and machined surface (right). **(C–F)** Biomechanical torsional evaluation results of machined titanium **(C–E)** and laser-treated titanium **(C,F)**. **(E)** TEM image of the laser-treated implant-bone interface. Reprinted with permission from (Palmquist et al., 2010).

**FIGURE 5:**
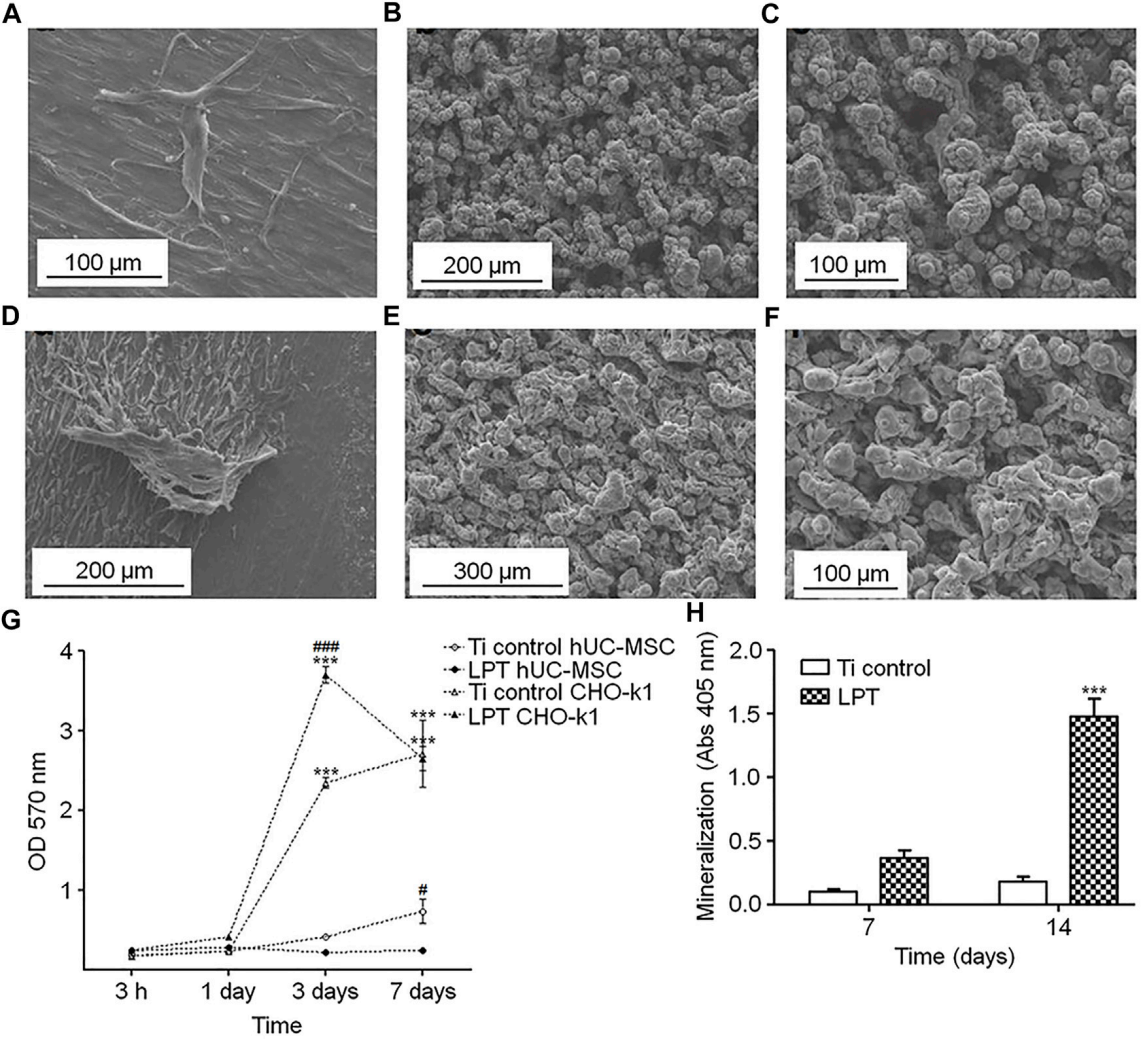
**(A–F)** hUC-MSCs on Ti control **(A)** and laser-processed titanium **(B,C)** surfaces. CHO-k1 cells on Ti control **(D)** and LPT **(E,F)** surfaces. **(G,H)** Results of cell metabolic activity assay and calcium deposition assay. Reprinted with permission from (Bressel et al., 2017).

Anders Palmquist et al. formed micro-nano surface topography and surface oxides on titanium screw valley through laser modification (Figures 4A,B). In biomechanical torsional evaluation, the laser-modified surface exhibited a high torque at breaking point and fractures away from the laser-treated surface (Figures 4C,F), while the machined surface had a low breaking point torque and showed fracture lines passing through or near the implant-bone interface (Figures 4C–E). The results indicates that laser modification improved the anchorage of the implant-bone interface (Figure 4G) (Palmquist et al., 2010). Research by Rickard Brånemark et al. also supports this result (Branemark et al., 2011). Human mesenchymal stem cells have been shown to play an important role in osteogenesis and osseointegration after implantation, but their differentiation ability decreases with age. Tatiana A. B. Bressel et al. loaded human umbilical cord mesenchymal stem cells (hUC-MSCs) with stronger differentiation ability and easier access on laser-processed titanium (LPT) (Figures 5A–F). Laser-modified surface inhibited the proliferation of hUC-MSCs (Figure 5G), but promoted the osteogenic differentiation of hUC-MSCs (Figure 5H). This shows the application prospects of hUC-MSCs-LPT biological scaffolds as orthopedic implants (Bressel et al., 2017).

**FIGURE 6:**
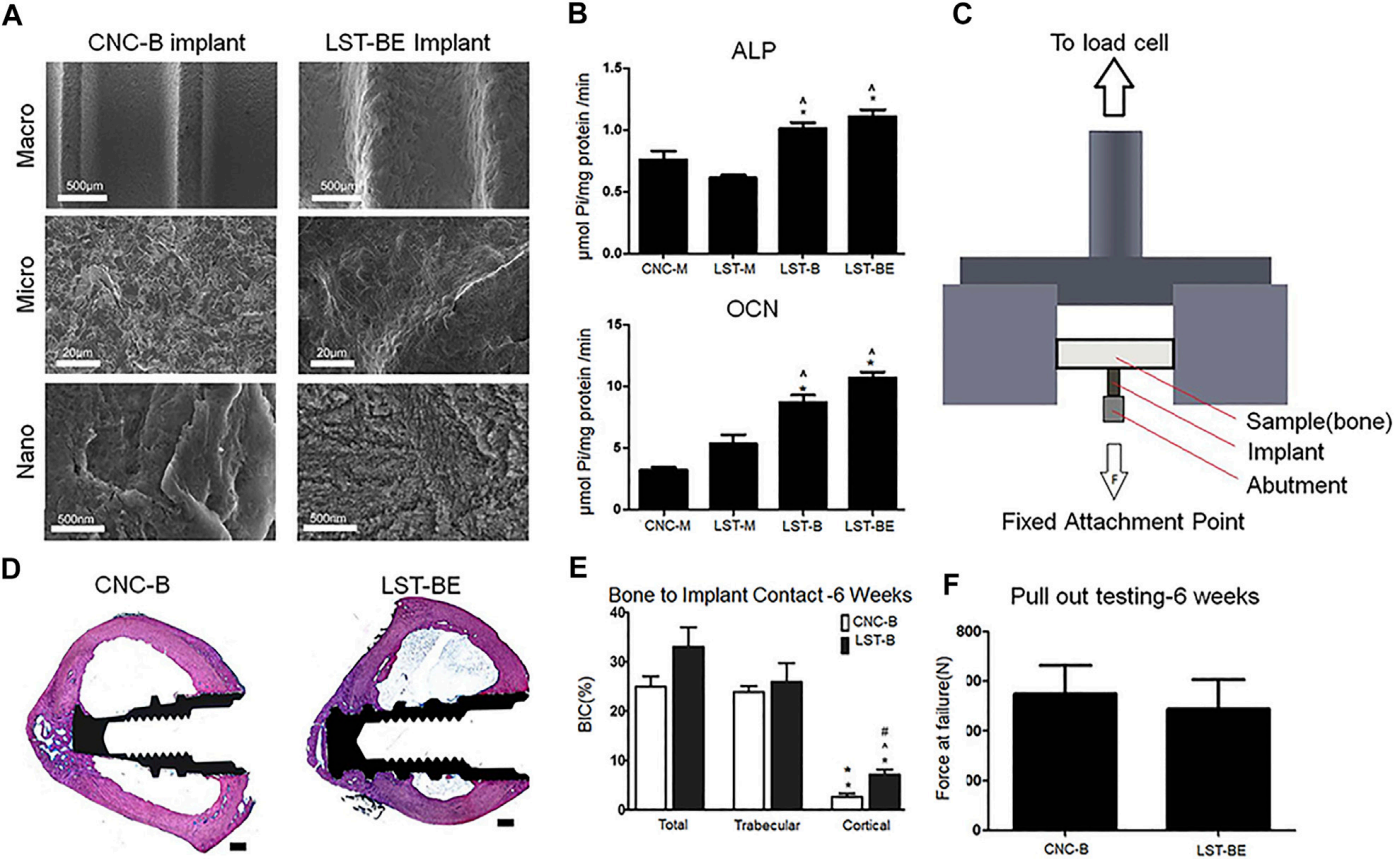
**(A)** SEM images of implant surfaces in different groups. **(B)** Quantitative results of osteogenic activity in different groups. **(C,F)** The schematic and results of pulling out mechanical testing of implants. **(D,E)** *In vivo*, Histological staining images and quantitative results of BIC. Reprinted with permission from (Hyzy et al., 2016).

Other processing can also be performed on the laser-modified titanium-based implant surface to further increase cell viability and osteogenic function. Sharon L. Hyzy et al. performed sand blasting and acid etching (BE) on the Ti-6Al-4V surface treated by direct metal laser sintering (DMLS) (Figure 6A), and found that the resulting meso-micro-nano rough surface (DMLS-BE) increased the osteoblast-like cell viability (Figure 6B). In experiments *in vivo*, the results showed that DMLS-BE material has similar mechanical stability to the computer numerical control-grit blasted (CNC-B) material used in clinical practice (Figure 6F), and exhibits better osseointegration, which indicates that DMLS- BE is a suitable substitute for traditional CNC-B (Figures 6D,E) (Hyzy et al., 2016). In addition, the disordered nanonet on SAH has a greater osteoinductive effect than the ordered nanotubes on SAN, which is consistent with the description in 3.1 section. Moreover, the disorderly arranged nanonet on SAH has the biomimetic characteristics similar to extracellular matrix (Xu et al., 2016).

## Hydrothermal Treatment

Hydrothermal treatment is a chemical processing method, carried out under high temperature and high pressure conditions, usually forming a thin coating on the surface of titanium-based implant, such as a layer of TiO_2_(Valanezhad et al., 2015; Zuldesmi et al., 2015; Yu et al., 2020; Huang et al., 2021). The processing method is simple, low-cost, and easy for industrial application. By adjusting the parameters of hydrothermal treatment, such as solution composition, temperature and treatment duration, different surface structures can be produced, such as nanoneedle, nanoscaffold, nanoplate, nanoleaf, and nanowire, etc., to promote cell proliferation, osteogenic differentiation and antibacterial effects (Son et al., 2003; Huang Y.-Z. et al., 2019).

In some studies, different nano-scale topographies were produced on the surface of titanium-based implant through hydrothermal treatment, which were characterized *in vivo* and *in vitro*. Qianli Huang et al. produced two different nanostructures, nanoplate and nanoleaf, by hydrothermal treatment for different time on the micropits surface produced by microarc oxidation. The adherent cells were round on the nanoplate surface, and elongated on the nanoleaf surface with deformation of the nucleus. Correspondingly, compared with the micropit, the nanoplate inhibited cell proliferation and had no obvious promotion effect on cell differentiation; while the nanoleaf had a promotion effect on cell proliferation and differentiation. This may be due to the fact that the nanoleaf provided a larger contact area for the adherent cells. In addition, among the three topographies, the nanoplate had the highest wettability, while the nanoleaf had the lowest wettability. This may indicate that the nano-scale topography of titanium-based surface plays a stronger role in enhancing cell response than wettability (Huang et al., 2016). V.V. Divya Rani et al. produced nanoneedle, nanoScaffold and nanoleaf on titanium implants through hydrothermal treatment, and also produced nanotube structures through anodizing treatment. The results showed that the nanoleaf showed the best function of promoting the osteoblasts proliferation and osseointegration in rats. In addition, nanoleaf induced changes in the cytoskeleton of osteoblasts, which in turn up-regulates the expression of certain genes, such as ALP, osteocalcin, collagen, decorin and Runx2 (Rani et al., 2012). The above researches show that the nanoleaf is a special nanostructure on titanium-based surface processed by hydrothermal treatment. Compared with other nanostructures, it shows better ability to promote cell proliferation, differentiation and bone formation.

**FIGURE 7:**
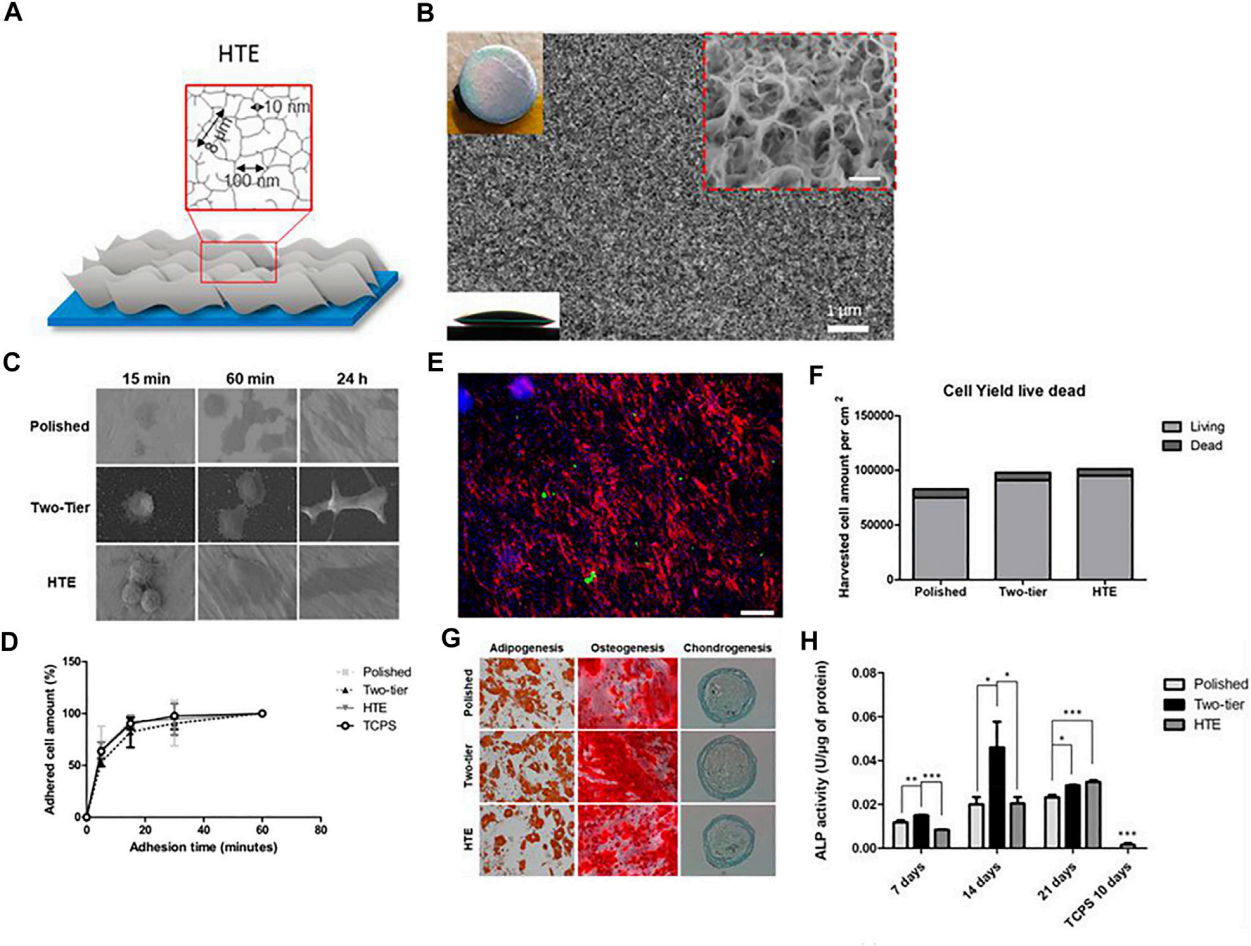
**(A,B)** Schematic representation and SEM image of hydrothermal treated titanium surface. **(C)** Different cell morphology on the surfaces over time. **(D)** Dynamic of cell attachment on different types of surfaces. **(E,F)** Live/dead cell staining images and cell viability after 96 h on hydrothermal treated Ti surface. Scale bars = 185 μm. **(G)** Staining images showing the differentiation ability of cells on different surfaces. **(H)** ALP activity of cells in different groups. Reprinted with permission from (Clainche et al., 2020). Copyright 2020, American Chemical Society.

Tristan Le Clainche et al. imitated the natural antibacterial surface of insect wings and produced random nanosheet structure on the Ti surface through a hydrothermal treatment (Figures 7A,B). This structure directly killed bacteria by cutting the bacterial cell membrane. As a nanostructure, it also retained the original adhesion, growth and proliferation ability of cells, and promoted osteogenic differentiation without the need for adding cytokines (Figures 7C–H) (Clainche et al., 2020). Martina Lorenzetti et al. proposed another antibacterial strategy. In their research, the titanium implant was coated with submicro-scale TiO_2_ structure through hydrothermal treatment. When the peak-to-peak spacing on the coating was less than the length of E. coli (about 2 μm), bacterial adhesion was reduced, otherwise it was increased (Lorenzetti et al., 2015). But further research on biosafety or osteoinductive function is needed.

## Electric Discharge Machining

Electric discharge machining (EDM), which is also called “spark erosion machining process,” is widely known as an unconventional surface treatment technique. While processing, the Tool and target samples are immersed in the dielectric. The surface material is removed by high-frequency pulsed sparks generated between the tool and workpiece, and the desired surface is finally formed. The processing principle of wire electric discharge machining (WEDM) is the same as that of EDM, and a metal guide wire is used as one side of the electrode. Due to the extremely thin wire, WEDM can finely produce complex structures.

For titanium and titanium alloys, traditional surface treatment methods are not ideal. Titanium and titanium alloys are considered difficult-to-cut materials due to their high strength (Ishfaq et al., 2020). In addition, the tools in traditional processing are easy to wear, which is mainly due to the thermal conductivity and chemical reactivity of titanium. The low thermal conductivity of titanium makes it hard to disperse the heat at the contact point during processing, resulting in local overheating and loss of the tool (Fuse et al., 2021). Titanium is easy to weld with tool at high temperature, making the tool invalid (Fuse et al., 2021).

The processing object of EDM is conductive material, and it can effectively process workpieces with high hardness, high strength and heat resistance. Therefore, EDM is developed as a new non-traditional treatment process for surface modification of titanium-based materials. The carbon and oxygen of the tool electrode and dielectric can be deposited on the material surface during processing to form a carbon- and oxygen-rich surface (Janecek et al., 2011; Strasky et al., 2011; Harcuba et al., 2012), which has been shown to enhance the response of bone cells. Surfaces produced by EDM are hydrophilic surfaces, which tend to favor cell adhesion and proliferation. In the study of Yamaki et al., the contact angles of the EDM surface and the machined surface without EDM were about 0° and 40°, respectively, and the new bone volume on the surface at 1 week after implantation was 38.7 and 6.9%, respectively. EDM significantly enhance early osteogenesis (Yamaki et al., 2012).

The peak current is the main factor determining the final surface roughness (Janecek et al., 2011). Increasing the peak current increases the surface roughness, which facilitates osseointegration after implantation, but also deteriorates the mechanical properties of the implant. In Petr et al.'s study, with increasing current (21–79 A), yield stress (∼775–∼725 MPa), ultimate tensile strength (∼850–∼800 MPa) and elongation (∼11.5–∼8%) generally decreased (Harcuba et al., 2012). Considering the dual effects of current on roughness and mechanical properties, 29 A is the optimal peak current (Harcuba et al., 2012). The study by Strasky et al. also came to the same conclusion (Strasky et al., 2011). A surface roughness Ra of 11.6 μm was obtained at this optimum current without significant adverse effects on mechanical properties (Janecek et al., 2011; Strasky et al., 2011; Harcuba et al., 2012).

However, low material removal rate (MRR) and tool wear are two issues that affect EDM applications, but can be partially improved by changing the dielectric composition and electrode material (Ishfaq et al., 2020). In addition, loose particles formed by resolidified metal droplets are common on EDM-processed surfaces, which risk shedding and damaging normal human tissue. These surface particles can be removed using a subsequent surface treatment process (Havlikova et al., 2014).

## Conclusion

This article reviews the application of several techniques for surface modification of titanium-based implants in orthopedics. For the surface modification of titanium-based implants, the following items are worth noting. First, with the rapid development of the concept of bionics in recent years, people pay more attention to it in the field of material design, and its applications in surface modification research will continue to increase (Chen et al., 2021; Li et al., 2021). Moreover, 3D printing, bioactive nanoparticles and stimulus-dependent variable materials have been research hotspots in recent years, and their combination with surface modification is expected to produce more new orthopedic implants. In addition, it is a trend of surface modification to produce composite surfaces structures with multiple morphologies and sizes. On the other hand, at present, researches on the effect of different shapes or scales on the surface of titanium-based plants in the healing process are not detailed and systematic enough. In addition, more researches on the mechanism are needed to guide the surface design of implants. The characteristics of the ideal surface-modified orthopedic implants in the future: mechanical properties close to that of bone, ability to promote bone healing, antibacterial ability, sufficient service life, easy to remove without complications, individualization, and highly automated and environmentally friendly treating process.
